# Choristoma: Cervical Chondrocutaneous Branchial Remnants

**DOI:** 10.7759/cureus.3149

**Published:** 2018-08-15

**Authors:** Matthew T Carvey, Devan Ramachandran, Robert Hage

**Affiliations:** 1 Medicine, St. George’s University, Grenada, WI, St. Georges, GRD; 2 School of Medicine, St. George's University, St. Georges, GRD; 3 Anatomical Sciences, St. George's University School of Medicine, St. Georges, GRD

**Keywords:** unilateral cervical chondrocutaneous branchial remnant, choristoma, right sternocleidomastoid

## Abstract

Unilateral cervical chondrocutaneous branchial remnants (CCBRs) are rare, but when present, are typically located over the lateral aspect of the neck along the anterior margin of the sternocleidomastoid muscle. A CCBR in this location is called a choristoma. Here, we describe a choristoma in a 25-year-old female who disclosed a documented diagnosis of Meniere’s disease, and an expressed interest in bearing children within the immediate future. She presented with a unilateral swelling, located subcutaneously, midway over the anterior margin of the right sternocleidomastoid muscle. Due to her history, and the risk of possible radiological exposure to her fetus, an ultrasound-based examination of the neck and cardio-abdomino-pelvic organs was performed. Our clinical findings, details of the ultrasound results, and surgical data will be described.

## Introduction

Cervical chondrocutaneous branchial remnants (CCBRs), being present both unilaterally or bilaterally, are rare lesions of embryonic origin. Chondrocutaneous branchial remnant is the universally accepted term for the presence of heterotopic cartilage subcutaneously in the cervical region [1]. To date, less than 117 cases have been reported in the medical literature, and 34 cases were bilateral [2]. The true origin of these lesions has been under scrutiny for the past century, and scarcity of reported cases hinders this process [3]. Histologically, a choristoma may appear to be of normal tissue type; however, it will be foreign to the location found on the specific organ in question [4]. They also consist of an elastic cartilage core covered by keratinizing squamous epithelium with skin appendages [5]. CCBRs are always benign, and vary markedly in their prevalence for the general population. Although CCBRs are rarely symptomatic, they may occasionally be associated with serious congenital anomalies. Therefore, identification of CCBRs can, and should, prompt further investigations.

Cervical chondrocutaneous branchial remnants located in the cervical region, particularly along the anterior border of the sternocleidomastoid muscle, are known as choristomas. By definition, a choristoma is a benign growth consisting of normal tissue located at an abnormal location. They may present as cysts, sinuses, fistulae, or cartilaginous remnants [6]. Theoretically the choristomas can be of different types such as heterotopic presence of thyroid gland, bone, glial tissue, and salivary gland [1]. Surgical excisions of these lesions are effective; however, swellings in the anterior neck along the sternocleidomastoid muscle carry a long list of differential diagnoses. Establishing a correct diagnosis is essential before treatment can be planned. 

## Case presentation

**Figure 1:**
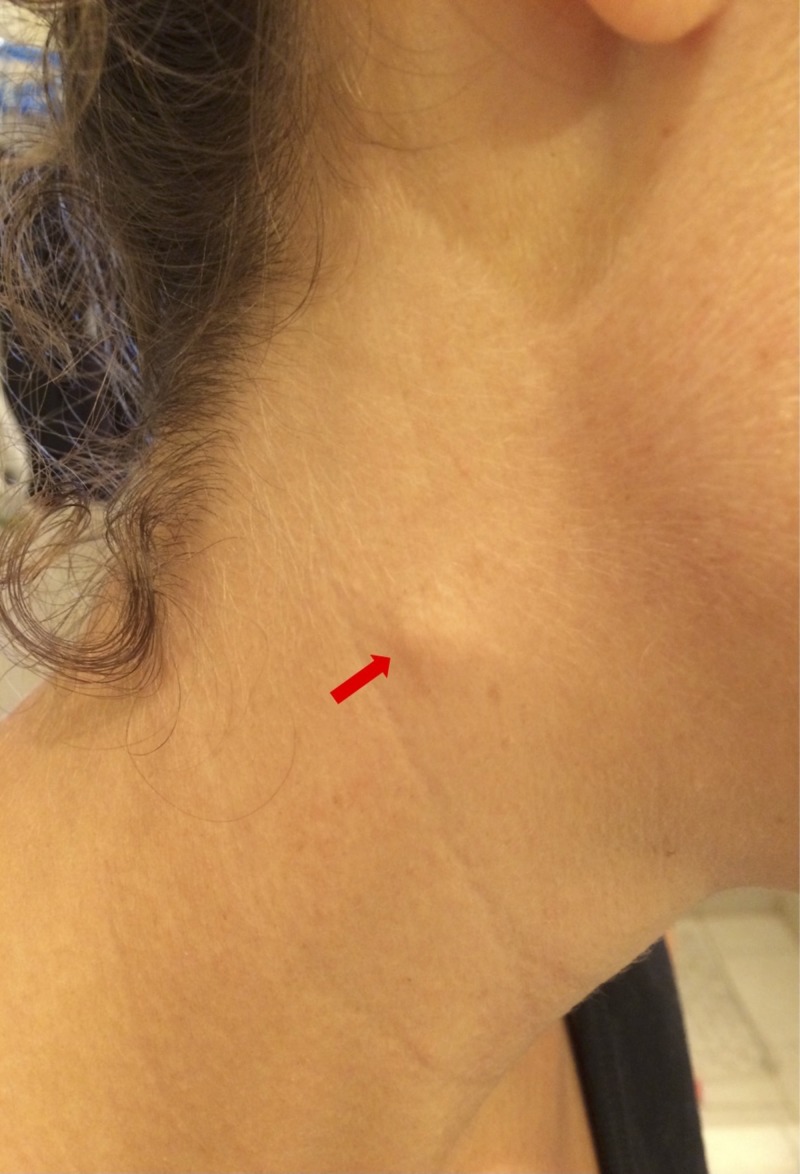
Swelling on the right sternocleidomastoid muscle, midway along its anterior border. The swelling was about 0.75 cm in length x 0.5 cm in width.

A 25-year-old woman temporarily living in Grenada visited a local otolaryngologist presenting with a past history of Meniere’s disease without treatment (based on a lack of active symptoms such as tinnitus, vertigo, and hearing loss). On presentation, she expressed that the motivation behind the visit was to investigate the significance of her neck swelling. She had missed her period for two consecutive months. Routine examination showed a swelling over the right sternocleidomastoid muscle, midway along its anterior border (Figure 1). The swelling was small, firm, subcutaneous, and partially attached to the skin. It had been present for as long as she could remember, with no associated symptoms. The history suggested a benign lesion consistent with the characteristics of a CCBR-choristoma.

Family history was unremarkable. No visible congenital anomalies such as aberrant implantation of the ears, auricular pits, auricular appendages, or fistulae were present. The swelling was about 0.75 cm in length x 0.5 cm in width. Palpation revealed a nontender structure in the subcutaneous plane unattached to the underlying tissue and mobile in every direction. The overlying skin was partially attached to the swelling, but it did not originate from within the skin (the skin above the swelling could be pinched up). No cervical lymph nodes draining the area were palpable. Examination of the left neck was unremarkable.

**Figure 2:**
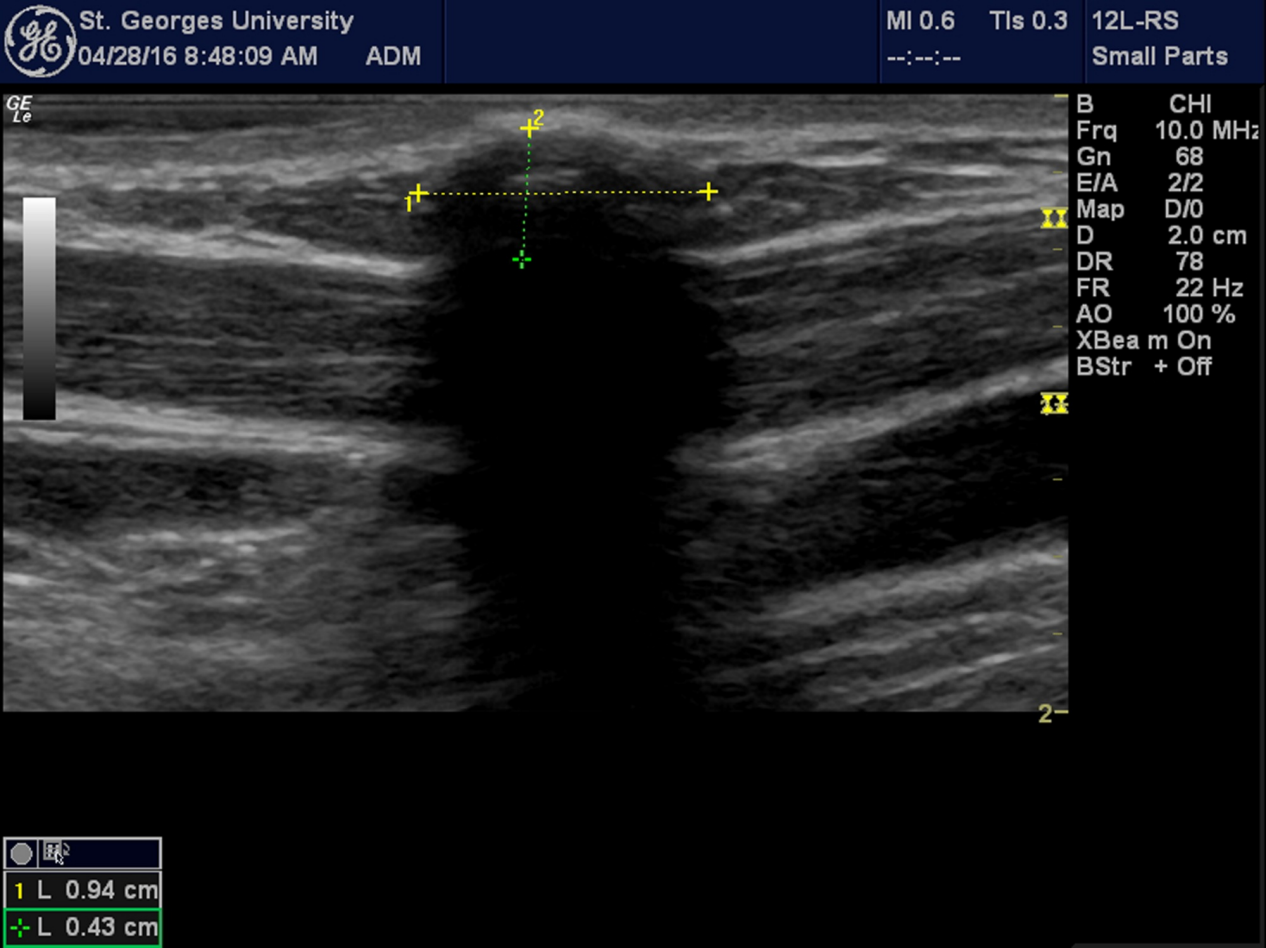
Ultrasound using a linear probe, showing a hypoechoic (likely cartilaginous) mass measuring 0.94 cm x 0.43 cm.

Due to her anxiety with respect to conceiving, and the risk of teratogenic radiological exposure, an ultrasound-based examination of the neck and cardio-abdomino-pelvic organs was performed to identify if her CCBR had associated defects. Ultrasound showed a hypoechoic (likely cartilaginous) mass measuring 0.94 cm x 0.43 cm (Figure 2). Abdominal ultrasound and cardiac examination are recommended because of possible associated anomalies [7]. These anomalies must be taken into consideration, as there is marked variation in the reported prevalence of associated anomalies, ranging from 11% to 76% [8]. Thus ultrasound, being the least invasive diagnostic technique, while also serving the patient with maximum utility, was the modality of choice.

The patient did not request surgical excision, as she was asymptomatic with the lesion for the duration of her lifetime. She was told, and reassured, that the ultrasound examination showed a benign cartilaginous mass. The ultrasound of her heart, abdomen, and pelvic organs also showed no abnormalities. This diagnosis allowed the patient to feel some comfort after what she assumed could have affected the health of her baby.

## Discussion

Cartilaginous embryological remnants in the neck are rare, in contrast to the prevalence of accessory tragi, a fairly common finding compared to CCBRs [9]. Embryologically, the first and second pharyngeal arches give rise to the auricle and middle ear structures, which are initially located ventrally in the lower lateral neck. Later, these structures migrate cranially along the anterior border of the sternocleidomastoid muscle. Improper or partial migration can result in remnant tissues at the site of origin, leading to CCBRs [10]. It has also been suggested that the presence of pluripotent cells can give rise to cartilaginous remnants [4]. Presence of cartilage in the excised lesion strongly favors a second pharyngeal arch origin while the presence of hyaline suggests a more proximal cervical location [11].

Diagnosis of cartilaginous choristoma was made. Other possible reasons for a swelling in the neck in this presented case could be a thymic cyst, thyroglossal duct, branchial cleft cyst, pilomatricoma, or hamartoma (Table 1). Goldenhar, Treacher-Collins, and some other well-characterized syndromes may include cervical or pre-auricular remnants [12]. All included differential diagnoses were considered and ruled out on further investigation into each.

**Table 1:** Summary of the differential diagnoses and treatment options related to cervical chondrocutaneous branchial remnants.

Differential diagnosis	Treatment options
Thymic cyst	Surgical resection
Thyroglossal duct	Surgical resection and treatment of possible underlying infection with an appropriate antibiotic
Branchial cleft cyst	Surgical resection
Pilomatricoma	Surgical resection
Hamartoma	Observation and possible surgical resection

Hamartomas are described as excessive, focal over-growth of cells and tissues native to the organ in which it occurs [13]. As embryonic development occurs there are many migrating parts, and when a cervical cutaneous branchial remnant persists it is more commonly a heterotopic rest or a choristoma; terms applied to microscopically normal cells or tissues that are present in abnormal locations [13].

A pilomatricoma is an abnormal swelling which is commonly located on the neck or head. However, on physical examination pilomatricomas are identified commonly by either or both the tent sign and the teeter-tot sign [14]. The tent sign is viewed by stretching the skin around the protrusion and the teeter-tot sign is examined by pushing on one half of the lesion and viewing the protrusion of the opposite half [14]. Neither the teeter-tot sign nor the tent sign was seen on examination, making a CCBR the more likely diagnosis.

The other differentials are possibilities, however, were overshadowed by the final diagnosis of CCBR due to all the facts presented within the case.

Treatment is complete surgical removal as promptly as possible to get an exact histopathological diagnosis [15]. If the patient involved is a pediatric, operative treatment can be postponed to a suitable and safe age [8]. Histopathological studies are then recommended, where investigations define this lesion as heterotopic, composed of normal skin and fat with a strip of cartilage running through the middle [16].

## Conclusions

A choristoma is a CCBR located specifically in the cervical region near the sternocleidomastoid muscle, particularly, a benign swelling that is firm on palpation and found subcutaneously. The choristoma in this case was present since birth on the right sternocleidomastoid muscle, had no size changes, was never infected, and not painful. This, with the assistance of ultrasound further confirming a hypoechoic cartilaginous mass, allowed the diagnosis of a choristoma to be made in this case. Other differential diagnoses were ruled out on further investigation.
